# Factors Associated with Dietary Behaviour During Pregnancy: An Insight from the *SaperePer* Cohort

**DOI:** 10.3390/nu18172939

**Published:** 2026-09-07

**Authors:** Samar El Sherbiny, Valeria Bellisario, Emilie Marion Canuto, Andrea Puppo, Luca Marozio, Roberto Bono

**Affiliations:** 1 Department of Public Health and Pediatrics, University of Turin, 10126 Turin, Italy; samar.elsherbiny@unito.it (S.E.S.); valeria.bellisario@unito.it (V.B.); roberto.bono@unito.it (R.B.); 2 Gynecology and Obstetrics 1U, Sant’Anna Hospital, AOU Città della Salute e della Scienza di Torino, 10126 Turin, Italy; emcanuto@cittadellasalute.to.it; 3 S.C. Obstetrics and Gynecology, AO Santa Croce e Carle, 12100 Cuneo, Italy; puppo.a@ospedale.cuneo.it; 4 Department of Surgical Sciences, Sant’Anna Hospital, University of Torino, 10126 Turin, Italy

**Keywords:** nutritional knowledge, public health, pregnancy, health promotion

## Abstract

Background: Diet during pregnancy plays a crucial role in promoting healthy dietary behaviours and positive maternal and child health outcomes. However, evidence regarding the factors associated with healthy dietary behaviours among pregnant women is limited. The *SaperePer* study aims to assess the dietary behaviours of pregnant women and identify the sociodemographic, lifestyle, and health-related factors that influence their choices. Methods: This cross-sectional analysis included 1254 pregnant women recruited from two Northern Italian centres (Turin and Cuneo). Dietary behaviour was assessed using a questionnaire, which generated a diet-related scores. Associations between these scores and participants’ characteristics were investigated using multivariable regression models. Results: Overall, participants reported relatively favourable dietary habits and a low prevalence of risky behaviours. Higher socioeconomic position was found to be one of the strongest contributing factors to dietary behaviour (medium vs. low β (95% CI) = 1.18 (0.93, 1.42); high vs. low β (95% CI) = 1.57 (1.10, 2.04), both *p* < 0.001). Higher dietary behaviour scores were positively associated with older maternal age, moderate physical activity, and better perceived physical and psychological health, whereas smoking and alcohol consumption were associated with lower scores. Conclusions: Dietary behaviour during pregnancy appears to be shaped by a complex interplay of socioeconomic, behavioural, and health-related factors rather than by nutritional knowledge alone. These findings highlight the need for tailored interventions that address the social and contextual features of dietary behaviour to encourage healthier habits during pregnancy.

## 1. Introduction

Food literacy has become a crucial intervention for the prevention of non-communicable diseases which represent a significant burden on the healthcare system. Health literacy refers to the set of abilities that enables individuals to access, understand, and appropriately use information related to their health to make informed decisions and maintain overall well-being [1]. People with adequate health literacy are able not only to locate reliable health information but also to interpret and critically assess it before applying it in everyday contexts [2]. This competence represents an important pillar of public health, as it supports disease prevention and contributes to limiting the burden of chronic communicable and non-communicable conditions, such as the aforementioned cardiovascular diseases, as well as diabetes and several forms of cancer, which are often closely linked to modifiable lifestyle behaviours [3]. Health literacy is a multidisciplinary construct encompassing domains such as the understanding of healthcare information, digital and media literacy, and mental health literacy [4]. Strengthening health literacy across populations is therefore essential to promote healthier choices and to foster empowerment in managing personal and community health. Health is particularly important among vulnerable groups, such as pregnant women, who face an extremely sensitive period during which they are highly motivated to engage with health information for the benefit of both themselves and their unborn child. Poor health literacy during pregnancy has been associated with poorer health outcomes and unhealthy behaviours, including inadequate diet and lifestyle habits. Among the different domains of health literacy, nutrition-related knowledge and skills have gained increasing attention due to their potential role in supporting healthier dietary choices [5]. During pregnancy, dietary behaviours are particularly relevant because maternal diet contributes to meeting increased nutritional requirements and may influence both maternal well-being and foetal development. Identifying factors associated with healthier dietary behaviours may therefore provide useful information for the design of targeted health promotion strategies [6,7,8,9,10]. Strengthening both health literacy and nutritional knowledge across pregnancy is essential to empower individuals to make informed choices and to reduce the potential health risks, such as cardiovascular disease or obesity, ultimately supporting more effective prevention strategies through improved knowledge and awareness [11]. The present study represents the first analytical component of the broader “*SaperePer*” project. While the overall project investigates pregnant women’s knowledge across multiple domains relevant to maternal and foetal health, including prenatal screening, this work specifically focuses on dietary behaviour as a distinct research objective. By concentrating on this single dimension, the study provides an in-depth and methodologically rigorous assessment of the factors associated with dietary behaviour, while preserving the standardised design and comprehensive methodological framework of the larger “*SaperePer*” project. Within the “*SaperePer*” study, designed to assess pregnant women’s knowledge on key topics such as prenatal screening, correct dietary behaviour during pregnancy was investigated as a secondary outcome. These outcomes are analysed alongside general information, including socioeconomic status, education, occupation, and overall health, to identify factors associated with dietary behaviour and to support the development of tailored health-promoting tools that facilitate informed decision-making for both maternal and foetal health. Counselling has been shown to increase awareness and improve health outcomes, particularly among women with lower educational or cultural resources [12]. Although food literacy represents a multidimensional construct, dietary behaviours constitute one of its behavioural manifestations and provide useful information on the adoption of healthy eating practices during pregnancy [13]. Overall, this study aims to investigate the factors associated with dietary behaviour among pregnant women within the “*SaperePer*” cohort, providing the first evidence from this broader project and generating findings that will inform the subsequent development of targeted health promotion interventions aimed at improving dietary behaviours and maternal and foetal health outcomes.

## 2. Materials and Methods

Following approval from the Bioethics Committee at the University of Turin (Prot. n. 0437754, 19 July 2024), recruitment took place at the Sant’Anna Hospital (Centre 1), specifically at the prenatal diagnostic birth centre (Turin, Piedmont), and at the Santa Croce Hospital in Cuneo (Centre 2). Pregnant women attending the study centres were invited to participate in the survey. There were no specific exclusion criteria. The questionnaire comprises four different sections, for a total of 53 questions, integrating different standardised questionnaires, including the 20-item Prenatal Health Behaviour Scale (PHBS). This self-report questionnaire uses a 5-point scale ranging from 0 (never) to 4 (always) to assess health-related behaviours during pregnancy, including smoking, diet, prenatal vitamin use, sleep and physical activity during the previous two weeks [14,15]. Of these, 10 questions pertained to the overall health status, and 10 questions addressed the dietary habits and lifestyle. Of the 10 diet-related questions, 6 were used to calculate a diet score on a scale between −6 and +6 to evaluate the diet quality of the participants. A score of 1 was given to the positive answers (answers 3 and 4), a score of −1 (answers 0 and 1) was given to negative answers in terms of healthy behaviours and 0 was given when the answer was neutral (answer 2). The remaining 4 items concerned beverages and supplements (e.g., water, coffee, alcohol, and supplements) and were not included in the score, to focus solely on food-related habits. As this was designed as a pilot exploratory analysis conducted on a subset of the overall study sample, this scoring approach was adopted to explore the potential of the selected diet-related items as independent indicators, rather than applying the standardised PHBS scoring system. The Italian version of the PHBS questionnaire and the proposed diet score will subsequently undergo formal validation as part of the broader project.

Socioeconomic status (SES) was calculated according to the Kuppuswamy scale [16]. Parity was assessed using a single-choice question, with participants assigned to one mutually exclusive category. The analyses were restricted to questionnaires completed in Italian in order to ensure dataset homogeneity and to minimise potential linguistic and cultural interpretation bias, which could have affected respondents’ understanding of the items and, consequently, the accuracy and comparability of the reported answers. No restrictions were applied in terms of gestational age to take part to the study.

### Data Analysis

Statistical analyses were performed using SPSS version 30.0.0.0 (IBM Corp., Armonk, NY, USA) and R version 4.4.3 (28 February 2025 ucrt; R Foundation for Statistical Computing, Vienna, Austria). Continuous variables were reported as means and standard deviations, while categorical variables were summarised as frequencies and percentages. The SES score was calculated using three scores: education (3 categories), employment status (2 categories), and household income (4 categories), resulting in a total SES score ranging from 3 to 9. Participants were classified into three SES groups based on the total score: low SES (3–4), middle SES (5–7), and high SES (8–9). This classification was based on the modified Kuppuswamy calculation method, with maternal personal income acting as a proxy for household income [17,18,19]. Regarding the other variables analysed, urbanisation was classified as cities (densely populated areas)/towns and suburbs (intermediate density areas)/rural areas (thinly populated areas) [20,21]. Age was then analysed as a dichotomous variable, in accordance with the clinical definition used to identify higher-risk pregnancies, distinguishing between women aged < 35 years and those aged ≥ 35 years [22]. The normality of the scores derived from the diet-related questions, and a second composite score combining diet and lifestyle factors (including smoking, alcohol consumption, sleep, physical activity and healthy eating habits), was assessed using the Shapiro–Wilk test, Q–Q plots, and evaluation of skewness and kurtosis. Both scores fell within acceptable ranges of normality. Depending on the type and distribution of the variables, independent-samples *t*-tests or Mann–Whitney U tests were used for comparisons between two groups, while one-way ANOVA or Welch’s ANOVA were used for comparisons among three or more groups. Linear regression models were used to examine the associations between diet score (dependent variable) and socio-demographic and pregnancy characteristics (maternal age, socioeconomic status [SES], urbanisation, and parity). A second fully adjusted model additionally included lifestyle- and health-related factors (physical activity, sleep, smoking status, alcohol consumption, self-perceived general health, and psychological health). Categorical variables with more than two categories were entered into the regression models using dummy coding, with the reference category specified a priori. Potential differences between study centres were formally assessed by including interaction terms between the study centre and each explanatory variable in the regression models. In addition, centre-stratified analyses were performed by fitting the same regression models separately within each study centre. Model assumptions were evaluated by assessing multicollinearity using generalised variance inflation factors (GVIF) and by inspecting residual diagnostics. As heteroscedasticity was detected using the Breusch–Pagan test, heteroscedasticity-consistent HC3 robust standard errors, 95% confidence intervals (95% CI), and *p*-values were reported. Effect estimates were expressed as regression coefficients (β) with corresponding 95% CI and *p*-values. Statistical significance was set at *p* < 0.05 (Table S1).

## 3. Results

### 3.1. Population

Population characteristics are presented in Table 1. Overall, 1254 pregnant women were included in the cohort and completed the questionnaire at Sant’Anna Hospital in Turin (n = 998) and Santa Croce e Carle Hospital in Cuneo (n = 256). The survey started in November 2024 and closed in June 2026. Most participants were younger than 35 years old (60.3%), and the majority resided in highly urbanised (43%) or moderately urbanised (44%) areas, while 13% lived in rural settings. A high educational level was observed in 64.4% of women, whereas only 0.8% had a low level of education. According to the socioeconomic status (SES) index, 76.6% of the cohort was classified as middle SES, followed by 18.3% as low SES and 5% as high SES. Regarding parity, 45.6% were nulliparous, 37.3% had at least one previous birth, and 17.1% reported a previous abortion; these data were not available for all participants. At the time of the survey, 61.5% of participants were in the first trimester, 23.3% in the second, and 15.2% in the third. Although the questionnaire was available in multiple languages, the present analysis included only participants who completed the Italian version. Overall, 18 participants (12 from Centre 1 and 6 from Centre 2) who completed the questionnaire in other languages were excluded from the analysis.

### 3.2. Diet and Lifestyle

Dietary and lifestyle behaviours in the full cohort generally reflected healthy patterns. Regarding dietary quality, as detailed in the Supplementary Material section (Table S1), most participants reported regular consumption of dairy and high-fibre foods, with 79.5% indicating a high frequency of intake. Consumption of junk food was low, with only 5.6% of women reporting frequent intake (similar across centres), while 51.6% reported never consuming such foods. Skipping meals or replacing them with snacks was uncommon, with 73.3% of participants reporting never doing so. In terms of quantity, 45.5% of the cohort reported “sometimes” eating excessively, and 9.4% reported doing so “always.” Conversely, adequate food intake was widely reported, with 85.6% stating they “always” ate enough. Balanced meals were also frequently consumed, with 80.3% of participants reporting “always,” with minimal differences between centres. Coffee consumption showed a more heterogeneous distribution: 37.8% reported “never,” 31.7% “sometimes,” and 30.5% “always” consuming coffee, with similar patterns across centres. Supplement use was common, with 65.6% reporting “always” taking supplements, while 19.9% reported no use. A difference between centres was observed: in Centre 2, nearly 30% of women reported no supplement use compared to 17.5% in Centre 1 (*p* < 0.001). Engagement in beneficial lifestyle behaviours was also high. Moderate physical activity was reported by 22.8% of participants, with a higher prevalence in Centre 1 (23%), while the majority of women reported engaging in both moderate and intense physical activity (35.5%), with a higher prevalence in Centre 2 (44%). Most participants (93.4%) reported getting enough sleep (≥6 h/night). Risk behaviours were relatively uncommon: 89.2% of women reported never having smoked, and alcohol consumption was even less frequent, with 91.9% reporting no alcohol intake during pregnancy. Finally, perceived health status was generally positive: 82.6% of participants rated their health as good, while only 3.3% reported poor health. A very similar result was observed for psychological health, with 81.7% of women reporting having good psychological health.

### 3.3. Dietary Behaviour Score

Diet score (Mean ± SD) for the total population was 3.9 ± 1.7. Table 2 represents diet score stratified (Table 2-PART A). Higher diet scores were observed among women aged > 35 years (*p* < 0.001 and *p* = 0.024, respectively), nulliparous women, those with higher socioeconomic status, women in a relationship, and participants born in Italy. A clear positive gradient was found across socioeconomic status categories, with scores increasing from low to high SES (both *p* < 0.001). Similarly, women born in Italy showed significantly higher diet scores than those born abroad (both *p* < 0.001). Significant differences were also observed according to age, parity, and marital status. The second part of Table 2 (Table 2-PART B) shows dietary scores by different habits, behaviours, and perceived health. A heterogeneous association was observed for physical activity, as women reporting only moderate physical activity exhibited the highest diet scores, whereas lower scores were observed among those reporting intense physical activity only, both moderate and intense physical activity, or no physical activity. Women who pursued generally good behaviours, such as getting enough sleep and not smoking or drinking, showed higher diet scores (all *p* < 0.001). Finally, diet scores were found to be higher in women with fair or high perceived physical and psychological health.

### 3.4. Factors Associated with Diet Score

A series of linear regression models were performed to identify and analyse factors able to potentially influence dietary behaviour among pregnant women. Two main models have been performed: (I) a sociodemographic model which evaluated the potential effects of age, SES, urbanisation and parity and (II) a lifestyle and health model which evaluated the potential effects of some modifiable lifestyle factors such as physical activity, sleep, smoking, alcohol consumption, together with perceived physical and psychological health during pregnancy. Analyses were stratified by study centre to explore potential differences across centres.

### 3.5. Sociodemographic Model

**Figure 1:**
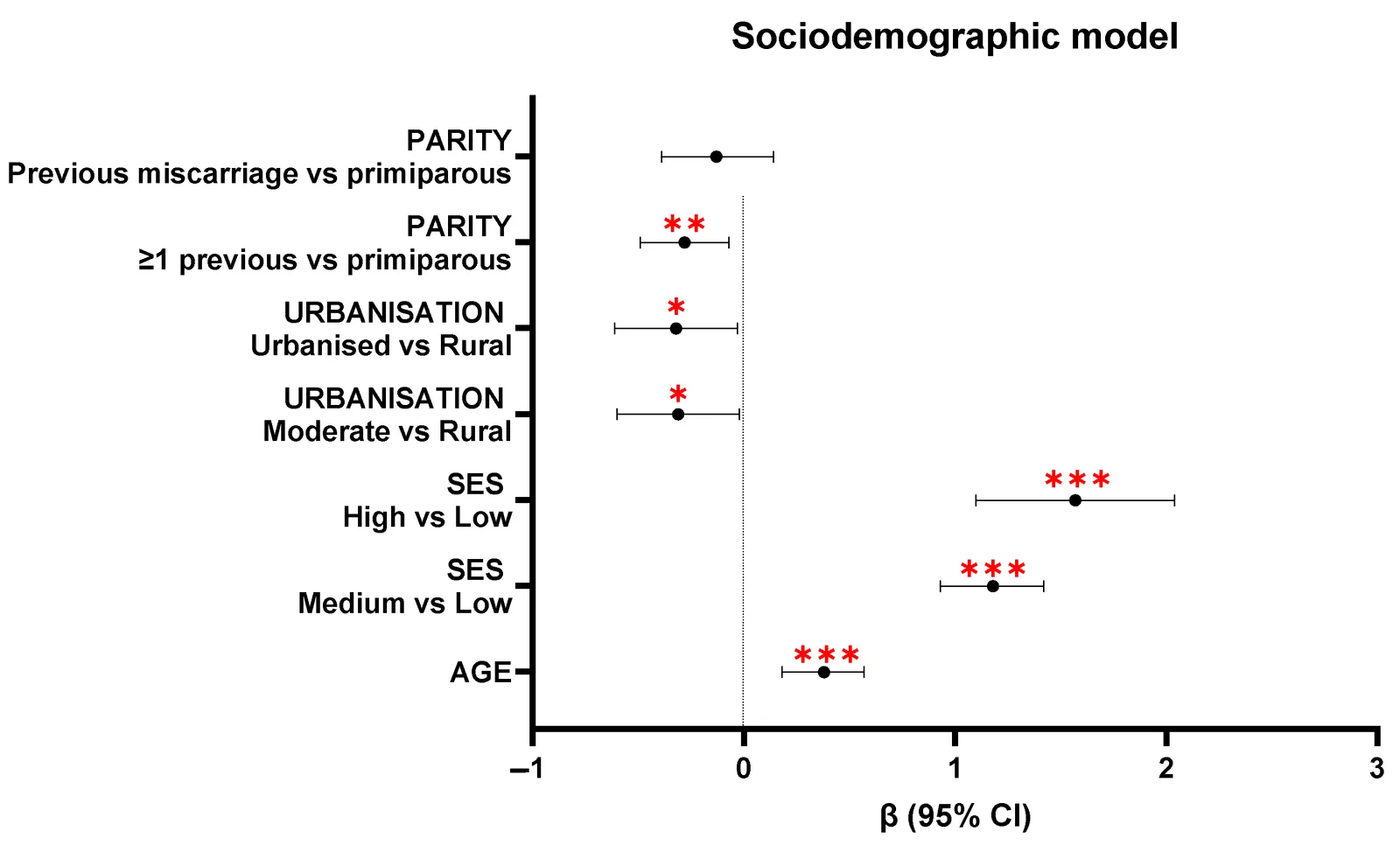
Forest plot of the sociodemographic model in the overall population based on the analyses reported in Table 3 (β (95% CI). * *p* < 0.05; ** *p* < 0.01; *** *p* < 0.001.

Table 3 and Figure 1 show results from the sociodemographic model, stratified per centre. In the overall model, which included both populations from Centre 1 and Centre 2, being older than 35 years was positively associated with a higher diet score compared with younger women (β (95% CI) = 0.38 (0.18, 0.57), *p* < 0.001). This association was consistent across both study centres. However, it reached statistical significance only in Centre 1 (β (95% CI) = 0.36 (0.14, 0.58), *p* = 0.001). SES was demonstrated to be one of the main predictors of diet score, with associations observed across both centres. In more detail, a higher SES strongly influenced diet-related knowledge (Table 3). Regarding urbanisation, both moderately urbanised and urbanised settings were associated with lower diet scores compared with rural residence in the overall model (β (95% CI) = 0.31 (−0.60, −0.02) and β (95% CI) = −0.32 (−0.61, −0.03), respectively, *p* = 0.034, and *p* = 0.029), with similar patterns observed in Centre 1. These associations were not statistically significant in Centre 2. Finally, parity was inversely associated with diet score in the overall sample, an association confirmed in Centre 1. However, in Centre 2 the association was positive, with women with previous children or a history of miscarriage having higher scores compared to primiparous women. Despite this inversion, these positive associations did not show statistical significance. Overall, associations between sociodemographic factors and diet score were broadly consistent across centres, with the exception of parity, which showed heterogeneous associations.

### 3.6. Lifestyle and Health Model

**Figure 2:**
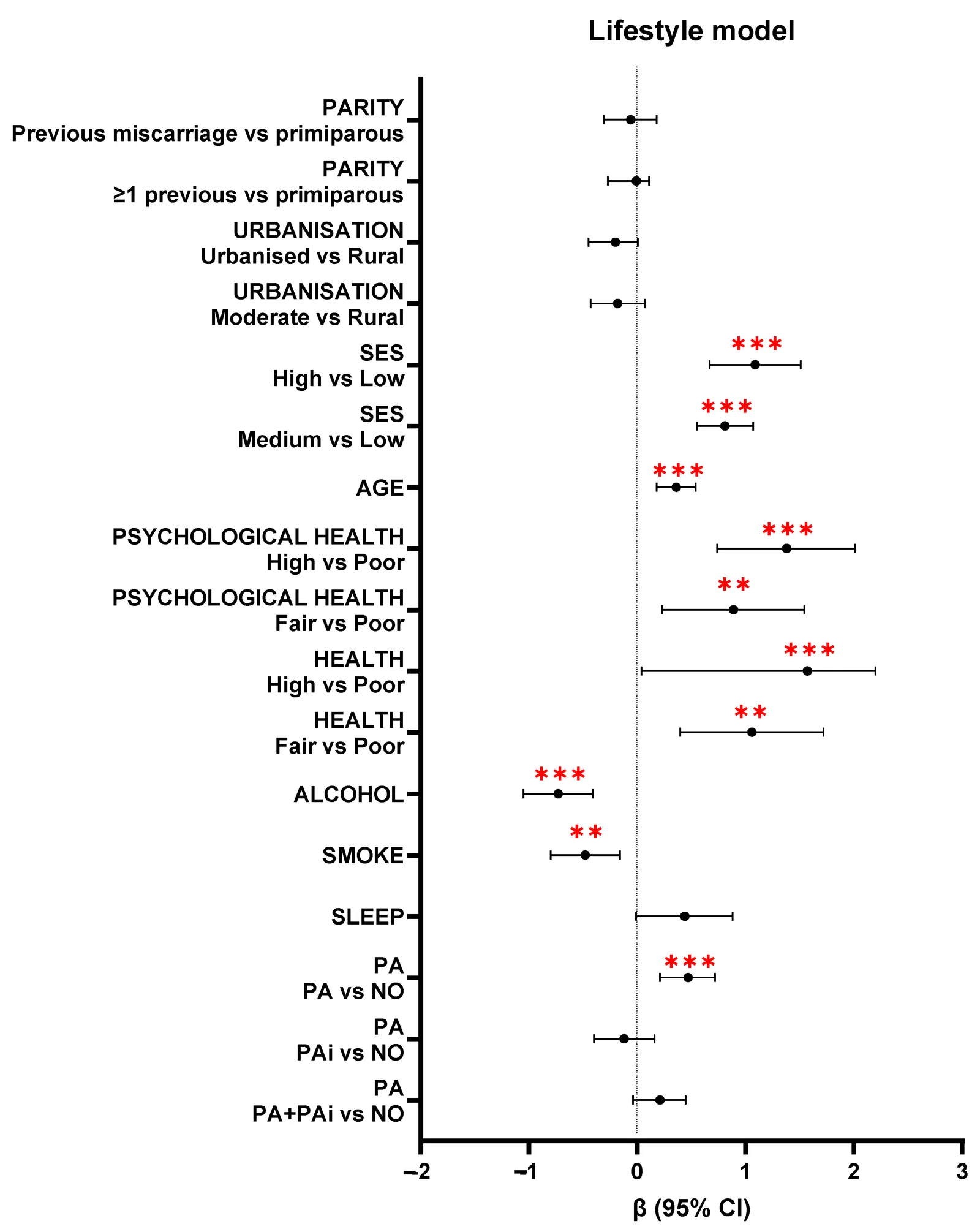
Forest plot of the lifestyle model in the overall population based on the analyses reported in Table 4 (β (95% CI). ** *p* < 0.01; *** *p* < 0.001.

Table 4 and Figure 2 present the results of the fully adjusted lifestyle and health model. The final model included 1164 complete observations (R^2^ = 0.261; adjusted R^2^ = 0.251). Overall, only moderate physical activity was positively associated with diet score. Compared with women reporting no physical activity, those engaging exclusively in moderate physical activity showed significantly higher diet scores in the overall sample (β (95% CI) = 0.47 (0.21, 0.72), *p* < 0.001), whereas the combined moderate-plus-vigorous activity category and vigorous activity alone were not significantly associated with the outcome. Similar estimates were observed in both study centres, although statistical significance was reached only in Centre 1. Adequate sleep duration (≥6 h/night) showed a positive but borderline association with diet score in the overall sample (β (95% CI) = 0.44 (−0.01, 0.88), *p* = 0.054), while no statistically significant associations were observed in the centre-specific analyses. Smoking and alcohol consumption were consistently associated with lower diet scores. Compared with non-smokers, smokers had significantly lower diet scores in the overall sample (β (95% CI) = −0.48 (−0.80, −0.16), *p* = 0.004) and in Centre 1, whereas the association was not statistically significant in Centre 2. Similarly, alcohol consumption was associated with lower diet scores in the overall sample (β (95% CI) = −0.73 (−1.05, −0.41), *p* < 0.001), and this association was consistently observed in both centres. Perceived physical and psychological health were also positively associated with diet score. Compared with women reporting poor general health, those reporting fair or good health had progressively higher diet scores in the overall model (β (95% CI) = 1.06 (0.40, 1.72), *p* = 0.002 and β (95% CI) = 1.57 (0.94, 2.20), *p* < 0.001, respectively). Likewise, women reporting fair and good psychological health achieved significantly higher diet scores than those reporting poor psychological health (β (95% CI) = 0.89 (0.23, 1.54), *p* = 0.008 and β (95% CI) = 1.38 (0.74, 2.01), *p* < 0.001, respectively). Similar trends were observed in Centre 1, whereas the corresponding associations in Centre 2 were attenuated and did not generally reach statistical significance. Overall, after adjustment for age, socioeconomic status, urbanisation, and parity, lifestyle and health-related characteristics remained independently associated with diet score, particularly moderate physical activity, smoking status, alcohol consumption, and perceived physical and psychological health.

## 4. Discussion

This study provides a comprehensive assessment of the factors associated with dietary behaviours among pregnant women, highlighting the contribution of both sociodemographic and modifiable lifestyle factors. Overall, our robust findings suggest that dietary behaviours during pregnancy are not solely associated with socioeconomic circumstances but also cluster with other health-promoting behaviours, supporting the concept that diet is embedded within broader healthy lifestyle patterns. Overall, participants reported relatively balanced dietary habits, while the prevalence of risk behaviours, such as smoking and alcohol consumption, was low, suggesting an overall favourable health profile in the studied population. Most previous studies have focused on nutritional knowledge, dietary behaviours, or health literacy more broadly, while fewer investigations have specifically explored the factors associated with food literacy in pregnant populations. Among the sociodemographic factors, socioeconomic status emerged as the strongest and most consistent variable associated with dietary behaviour across both study centres. Women with medium and high SES showed progressively higher diet scores than those with lower SES, indicating a clear socioeconomic gradient. This finding is consistent with previous evidence [23,24] demonstrating that socioeconomic resources facilitate access to nutrition information, healthier food environments, and greater opportunities to translate nutritional knowledge into everyday dietary practices [25,26]. Higher socioeconomic position has repeatedly been associated with greater health literacy, healthier dietary habits [23,27], and improved adherence to dietary recommendations during pregnancy, suggesting that food literacy and overall healthy dietary habits may represent one of the pathways linking social inequalities to maternal and child health outcomes [28]. Maternal age also showed a positive association with diet score, with women aged 35 years or older displaying significantly higher diet scores. Older maternal age has previously been associated with greater health awareness, increased engagement with prenatal care, and higher adherence to dietary recommendations as previously demonstrated in another Italian cohort [29]. A possible explanation of our observation can be that this association may reflect greater life experience and psychological readiness associated with advanced maternal age, which may contribute to greater awareness of health-related issues and engagement with healthy behaviours during pregnancy [30]. Parity showed a more complex pattern. In the overall cohort, women with previous pregnancies had slightly lower diet scores compared with nulliparous women, whereas the opposite trend, although not statistically significant, was observed in Centre 2. These findings may reflect different behavioural mechanisms. First-time mothers are often more engaged with antenatal education and may actively seek nutritional information due to the novelty of pregnancy, while multiparous women may rely more on previous experience and therefore engage less frequently with updated recommendations [31]. These factors could potentially contribute to differences in dietary behaviours according to parity, although the mechanisms underlying this association were not directly assessed in the present study. Conversely, previous pregnancy experience may also improve practical food-related skills, potentially explaining the opposite trend observed in the smaller centre [31,32]. The inconsistency across centres suggests that parity is likely context-dependent and influenced by different local characteristics, healthcare pathways, or cultural factors. An unexpected finding was the inverse association between urbanisation and diet score, with women living in rural areas showing slightly higher diet scores than those residing in urban or moderately urbanised settings. Although urban environments generally provide greater access to healthcare services, nutrition counselling, and healthy food outlets, they may also expose individuals to more obesogenic food environments [33,34], higher availability of ultra-processed foods [35,36], and time constraints that negatively influence dietary behaviours. Nevertheless, this association should be interpreted cautiously because differences were relatively small and were not consistently observed across both centres. One of the most relevant findings of this study is the close relationship between dietary behaviours and modifiable lifestyle behaviours. Women engaging in moderate physical activity demonstrated significantly higher diet scores, whereas intense physical activity alone was negative, but not significantly associated with the diet score. This finding aligns with current pregnancy guidelines, which recommend regular moderate-intensity exercise as part of a healthy lifestyle [37,38]. The positive association observed between moderate physical activity and diet score may be consistent with the clustering of health-related behaviours, whereby physically active women may also be more likely to adopt healthier dietary behaviours. However, this interpretation remains hypothetical, as patterns of health-related behaviour clustering were not directly assessed in the present study. The negative direction observed for intense physical activity may reflect differences in the characteristics or motivations of women engaging in higher-intensity activity; however, this possible explanation could not be directly evaluated in our study [39]. A positive association between adequate sleep duration and diet score was identified, although not statistically significant. Sleep is rarely investigated within food literacy research, but growing evidence indicates that insufficient sleep influences appetite regulation, food preferences, and dietary quality [40]. Adequate sleep may therefore represent both a marker and a facilitator of healthier lifestyle patterns during pregnancy [41], reinforcing the interconnected nature of health behaviours. Smoking and alcohol consumption, as well, were consistently associated with substantially lower diet scores. These findings suggest that dietary behaviours during pregnancy may cluster with other health-related behaviours, reflecting broader patterns of health practices rather than isolated dietary choices. Women who smoke or consume alcohol during pregnancy may also show lower adherence to preventive health recommendations, including dietary advice; however, this interpretation should be considered cautiously given the cross-sectional design of the study [42]. Finally, another noteworthy finding concerns the association between self-perceived physical and psychological health and diet score. Women reporting good general health or psychological wellbeing consistently achieved higher diet scores than those reporting poor health. Although cross-sectional design precludes causal inference, several mechanisms may explain this relationship. Again, better physical and mental health may facilitate healthier food choices through increased motivation [43], while healthier dietary patterns may contribute to improved wellbeing through psychological pathways Firth, et al. [44]. Recent qualitative evidence further suggests that maternal food literacy extends beyond nutritional knowledge and encompasses the ability to critically appraise information, manage uncertainty, and translate recommendations into everyday food-related decisions, processes that may themselves be influenced by emotional wellbeing [45,46].

### Strengths and Limitations

This study has several strengths. These include the relatively large sample size, the inclusion of women from two different healthcare settings, and the comprehensive assessment of social, demographic, health-related, and behavioural factors associated with dietary behaviour. Including women from two different healthcare settings also enabled the consistency of the observed associations across study centres to be examined. Nevertheless, several limitations should be acknowledged. Firstly, the cross-sectional nature of the analyses does not permit the establishment of causal relationships. Secondly, as all behavioural variables were self-reported, they may be subject to recall and social desirability bias, particularly regarding smoking, alcohol consumption, and dietary habits during pregnancy [47]. Thirdly, the study population was characterised by relatively high educational attainment and generally healthy lifestyle, which may limit the generalisability of the findings to more socioeconomically disadvantaged populations. Fourthly, the dietary behaviour/diet score used as the main outcome was derived from selected dietary items in the questionnaire. It should not be interpreted as a comprehensive measure of food literacy, which encompasses broader skills related to accessing, understanding, appraising, and applying nutrition-related information. Finally, although the analyses were stratified by study centre, formal interaction analyses did not identify statistically significant differences in the associations between the explanatory variables and the diet score according to study centre. Therefore, the observed differences in individual estimates between centres should be interpreted with caution, particularly given the smaller sample size of the Cuneo cohort and the consequently lower statistical power for some associations.

## 5. Conclusions

From a public health perspective, these findings have important implications. Pregnancy represents a unique window of opportunity during which women are highly motivated to adopt healthier behaviours and have frequent contact with healthcare professionals. The identification of groups at greater risk of lower healthy diet behaviour, including younger women, those with lower socioeconomic status, smokers, and women reporting poorer health, may facilitate targeted interventions integrated into routine antenatal care. Importantly, our results suggest that dietary behaviour during pregnancy is associated with broader lifestyle and health-related profiles. Therefore, future health promotion strategies could consider addressing dietary behaviours alongside other lifestyle domains, including physical activity, sleep, smoking cessation, and psychological wellbeing. Integrated approaches targeting multiple lifestyle factors may represent a promising area for future research; however, prospective and interventional studies are needed to evaluate their effectiveness in improving maternal dietary behaviours and related health outcomes.

## Figures and Tables

**Table 1:** Population characteristics of the *SaperePer* cohort in the full cohort and subgrouped per centre (Centre 1 = Turin; Centre 2 = Cuneo).

Characteristics	Full Cohort 1254 (100%)	Centre 1 998 (79.6%)	Centre 2 256 (20.4%)
Age N (%)			
<35 y.o.	756 (60.3)	565 (56.6)	191 (74.6)
>35 y.o.	498 (39.7)	433 (43.4)	65 (25.4)
Parity N (%)			
None	531 (45.6)	418 (44.8)	113 (48.9)
1 or more	434 (37.3)	334 (35.8)	100 (43.3)
Abortion	199 (17.1)	181 (19.4)	18 (7.8)
Education N (%)			
Low	10 (0.8)	7 (0.7)	3 (1.2)
Medium	436 (34.8)	328 (32.9)	108 (42.2)
High	808 (64.4)	663 (66.4)	145 (56.6)
SES N (%)			
Low	230 (18.3)	186 (18.6)	44 (17.2)
Middle	961 (76.6)	759 (76.1)	202 (78.9)
High	63 (5)	53 (5.3)	10 (3.9)
Urbanisation N (%)			
Rural	163 (13)	90 (9)	73 (28.5)
Moderately urbanised	552 (44)	408 (40.9)	144 (56.3)
Urbanised	539 (43.0)	500 (50.1)	39 (15.2)
Smoking			
Yes	136 (10.8)	106 (10.6)	30 (11.7)
No	1118 (89.2)	892 (89.4)	226 (88.3)
Alcohol consumption			
Yes	101 (8.1)	75 (7.5)	26 (10.2)
No	1153 (91.9)	923 (92.5)	230 (89.8)
Physical activity			
No PA	278 (22.2)	235 (23.5)	43 (16.8)
PA + PAi	445 (35.5)	332 (33.3)	113 (44.1)
PA	286 (22.8)	230 (23)	56 (21.9)
PAi	245 (19.5)	201 (20.1)	44 (17.2)
Sleep			
Yes	1171 (93.4)	932 (93.4)	239 (93.4)
No	83 (6.6)	66 (6.6)	17 (6.6)
Perceived health			
Bad	42 (3.3)	36 (3.6)	6 (2.3)
Fair	176 (14)	151 (15.1)	25 (9.8)
Good	1036 (82.6)	811 (81.3)	225 (87.9)
Psychological health			
Bad	45 (3.6)	39 (3.9)	6 (2.3)
Fair	184 (14.7)	151 (15.1)	33 (12.9)
Good	1025 (81.7)	808 (81)	217 (84.8)

SES = socioeconomic status; y.o. = years old; PA = physical activity; PAi = intense physical activity;

**Table 2:** (**A**): Diet score according to sociodemographic characteristics of the population. (**B**): Diet score according to different lifestyle categories.

(A)		
Characteristics	Diet Score (Mean ± SD)	*p*-value
Age		
<35 y.o.	3.7 ± 1.8	<0.001
>35 y.o.	4.2 ± 1.6	
Parity		
None	4.0 ± 1.5	0.020
1 or more	3.7 ± 1.8	
Abortion	3.9 ± 1.7	
SES		
Low	2.8 ± 2.0	<0.001
Middle	4.1 ± 1.5	
High	4.5 ± 1.4	
Marital status		
Single	2.8 ± 2.1	<0.001
Other	3.2 ± 2.4	
In a relationship	3.9 ± 1.7	
Urbanisation		
Rural	4.1 ± 1.5	0.115
Moderately urbanised	3.8 ± 1.7	
Urbanised	3.9 ± 1.8	
Birth country		
Italy	4.0 ± 1.7	<0.001
Other	2.9 ± 1.9	
Centre		
Centre 1	3.9 ± 1.7	0.376
Centre 2	3.9 ± 1.7	
(B)		
Characteristics	Diet score (Mean ± SD)	*p*-value
Physical activity		
No PA	3.58 ± 1.97	<0.001
Only PA	4.43 ± 1.42	
Only PAi	3.47 ± 1.70	
PA + PAi	3.91 ± 1.61	
Sleep		
Yes	3.9 ± 1.6	0.003
No	3.1 ± 2.4	
Smoking		
Yes	2.7 ± 1.9	<0.001
No	4.0 ± 1.7	
Alcohol		
Yes	2.9 ± 1.8	<0.001
No	4.0 ± 1.7	
General health		
Bad	1.4 ± 2.5	<0.001
Fair	3.1 ± 2.0	
Good	4.1 ± 1.5	
Psychological health		
Bad	1.5 ± 2.4	<0.001
Fair	3.3 ± 1.8	
Good	4.1 ± 1.5	

SD = standard deviation; SES = socioeconomic status; PA = physical activity; PAi = intense physical activity.

**Table 3:** Results from the sociodemographic model, stratified per centre.

Variable	Total Sample		Centre 1		Centre 2	
	β (95% CI)	*p*	β (95% CI)	*p*	β (95% CI)	*p*
Age						
≥35 y.o. vs. <35 y.o.	0.38 (0.18, 0.57)	<0.001	0.36 (0.14, 0.58)	0.001	0.45 (−0.09, 0.99)	0.100
SES						
Medium vs. low	1.18 (0.93, 1.42)	<0.001	1.21 (0.93, 1.48)	<0.001	1.06 (0.49, 1.63)	<0.001
High vs. low	1.57 (1.10, 2.04)	<0.001	1.51 (1.00, 2.02)	<0.001	1.98 (0.70, 3.27)	0.003
Urbanisation						
Moderately urbanised vs. rural	−0.31 (−0.60, −0.02)	0.034	−0.39 (−0.76, −0.01)	0.043	−0.13 (−0.61, 0.34)	0.580
Urbanised vs. rural	−0.32 (−0.61, −0.03)	0.029	−0.34 (−0.71, 0.03)	0.072	−0.55 (−1.21, 0.11)	0.103
Parity						
≥1 previous pregnancy vs. primiparous	−0.28 (−0.49, −0.07)	0.008	−0.40 (−0.63, −0.16)	0.001	0.13 (−0.33, 0.60)	0.572
Previous miscarriage vs. primiparous	−0.13 (−0.39, 0.14)	0.351	−0.24 (−0.52, 0.04)	0.092	0.66 (−0.15, 1.47)	0.108

SES = socioeconomic status; CI = confidence interval.

**Table 4:** Results from the lifestyle and health model stratified per centre.

Variable	Total Sample		Centre 1		Centre 2	
	β (95% CI)	*p*	β (95% CI)	*p*	β (95% CI)	*p*
PA						
PA + PAi vs. none	0.21 (−0.04, 0.45)	0.095	0.21 (−0.06, 0.48)	0.130	0.31 (−0.32, 0.94)	0.340
Only PAi vs. none	−0.12 (−0.40, 0.16)	0.383	−0.13 (−0.44, 0.18)	0.409	−0.05 (−0.77, 0.66)	0.881
Only PA vs. none	0.47 (0.21, 0.72)	<0.001	0.45 (0.18, 0.73)	0.001	0.50 (−0.17, 1.18)	0.147
Sleep						
Adequate sleep vs. inadequate	0.44 (−0.01, 0.88)	0.054	0.40 (−0.10, 0.89)	0.116	0.48 (−0.70, 1.67)	0.423
Smoking						
Smokers vs. non-smokers	−0.48 (−0.80, −0.16)	0.004	−0.58 (−0.95, −0.22)	0.002	−0.01 (−0.65, 0.63)	0.976
Alcohol consumption						
Alcohol consumption vs. no alcohol	−0.73 (−1.05, −0.41)	<0.001	−0.61 (−0.99, −0.23)	0.002	−1.29 (−1.99, −0.58)	<0.001
General health						
Fair vs. poor	1.06 (0.40, 1.72)	0.002	0.95 (0.21, 1.69)	0.013	1.68 (−0.30, 3.66)	0.098
Good vs. poor	1.57 (0.94, 2.20)	<0.001	1.50 (0.78, 2.22)	<0.001	1.80 (0.04, 3.56)	0.046
Psychological health						
Fair vs. poor	0.89 (0.23, 1.54)	0.008	0.88 (0.18, 1.59)	0.014	0.75 (−1.53, 3.02)	0.521
Good vs. poor	1.38 (0.74, 2.01)	<0.001	1.36 (0.68, 2.04)	<0.001	1.37 (−0.85, 3.59)	0.229
Age						
≥35 y.o. vs. <35 y.o.	0.36 (0.18, 0.54)	<0.001	0.33 (0.13, 0.52)	0.001	0.58 (0.05, 1.12)	0.035
SES						
Medium vs. low	0.81 (0.55, 1.07)	<0.001	0.84 (0.55, 1.13)	<0.001	0.77 (0.12, 1.42)	0.022
High vs. low	1.09 (0.67, 1.51)	<0.001	1.07 (0.61, 1.53)	<0.001	1.26 (0.14, 2.38)	0.028
Urbanisation						
Moderately urbanised vs. rural	−0.18 (−0.43, 0.07)	0.158	−0.25 (−0.56, 0.05)	0.106	−0.06 (−0.50, 0.37)	0.782
Urbanised vs. rural	−0.20 (−0.45, 0.06)	0.127	−0.23 (−0.53, 0.07)	0.126	−0.47 (−1.25, 0.31)	0.236
Parity						
≥1 previous pregnancy vs. primiparous	−0.08 (−0.27, 0.11)	0.423	−0.17 (−0.39, 0.05)	0.133	0.29 (−0.16, 0.74)	0.215
Previous miscarriage vs. primiparous	−0.06 (−0.31, 0.18)	0.610	−0.15 (−0.41, 0.11)	0.271	0.51 (−0.30, 1.32)	0.219

PA = physical activity; PAi = intense physical activity; CI = confidence interval.

## Data Availability

The datasets generated and analysed during the current study are available by request.

## Supplementary Materials

The following supporting information can be downloaded at https://www.mdpi.com/article/10.3390/nu18172939/s1, Table S1: Lifestyle and health characteristics of the *SaperePer* cohort. Table S2. Interaction terms between study centre and lifestyle- and health-related variables in the fully adjusted regression model.

## Abbreviations

The following abbreviations are used in this manuscript:

SES	socioeconomic status
y.o.	years old
PA	physical activity
PAi	intense physical activity
SD	standard deviation
CI	confidence interval
