# Supplementary material for: Factors Associated with Dietary Behaviour During Pregnancy: An Insight from the SaperePer Cohort
Source: Nutrients. 2026 Sep 7;18(17):2939. doi: 10.3390/nu18172939 (PMC13567649; doi:10.3390/nu18172939)
Supplement: Supplementary file 1 [file nutrients-18-02939-s001.zip › nutrients-4468317-supplementary.pdf]

SUPPLEMENTARY MATERIALS

Table S1. Lifestyle and health characteristics of the SaperePer cohort.

Characteristics	Full Cohort 1254 (100%)	Centre 1 998(79.6%)	Centre 2 256(20.4%)
**Dairy and high fiber food consumption**			
**Never**	41(3.3)	30(3)	11(4.3)
**Sometimes**	216(17.2)	164(16.4)	52(20.3)
**Always**	997(79.5)	804(80.6)	193(75.4)
**Junk food consumption**			
**Never**	647(51.6)	518(51.9)	129(50.4)
**Sometimes**	537(42.8)	421(42.2)	116(45.3)
**Always**	70(5.6)	59(5.9)	11(4.3)
**Meal skipping or snack substitution**			
**Never**	919(73.3)	738(73.9)	181(70.7)
**Sometimes**	285(22.7)	219(21.9)	66(25.8)
**Always**	50(4)	41(4.1)	9(3.5)
**Eaten too much food**			
**Never**	566(45.1)	435(43.6)	131(51.2)
**Sometimes**	570(45.5)	468(46.9)	102(39.8)
**Always**	118(9.4)	95(9.5)	23(9)
**Eaten enough food**			
**Never**	40(3.2)	35(3.5)	5(2)
**Sometimes**	140(11.2)	112(11.2)	28(10.9)
**Always**	1074(85.6)	851(85.3)	223(87.1)
**Balanced and healthy meal**			
**Never**	40(3.2)	34(3.4)	6(2.3)
**Sometimes**	207(16.5)	163(16.3)	44(17.2)
**Always**	1007(80.3)	801(80.3)	206(80.5)
**Coffee consumption**			
**Never**	474(37.8)	380(38.1)	94(36.7)
**Sometimes**	398(31.7)	307(30.8)	91(35.5)
**Always**	382(30.5)	311(31.2)	71(27.7)
**Supplements**			
**Never**	249(19.9)	175(17.5)	74(28.9)
**Sometimes**	183(14.6)	143(14.3)	40(15.6)
**Always**	822(65.6)	680(68.1)	142(55.5)
**Smoking**			
**Yes**	136(10.8)	106(10.6)	30(11.7)
**No**	1118(89.2)	892(89.4)	226(88.3)
**Alcohol consumption**			
**Yes**	101(8.1)	75(7.5)	26(10.2)
**No**	1153(91.9)	923(92.5)	230(89.8)
**Physical activity**			
**No PA**	278(22.2)	235(23.5)	43(16.8)
**PA+PAi**	445(35.5)	332(33.3)	113(44.1)

**PA**	286(22.8)	230(23)	56(21.9)
**PAi**	245(19.5)	201(20.1)	44(17.2)
**Sleep**			
**Yes**	1171(93.4)	932(93.4)	239(93.4)
**No**	83(6.6)	66(6.6)	17(6.6)
**Perceived health**			
**Bad**	42(3.3)	36(3.6)	6(2.3)
**Fair**	176(14)	151(15.1)	25(9.8)
**Good**	1036(82.6)	811(81.3)	225(87.9)
**Psychological health**			
**Bad**	45(3.6)	39(3.9)	6(2.3)
**Fair**	184(14.7)	151(15.1)	33(12.9)
**Good**	1025(81.7)	808(81)	217(84.8)

Table S2. Interaction terms between study centre and lifestyle- and health-related variables in the fully adjusted regression model

**Variable**	**Interaction term (Centre × Variable) β (95% CI)**	**p-value**
Physical activity		
PA + vigorous PA vs none	-0.03 (-0.64, 0.58)	0.927
Vigorous PA only vs none	0.09 (-0.63, 0.80)	0.828
Moderate PA only vs none	-0.04 (-0.72, 0.64)	0.917
Sleep		
Adequate vs inadequate	0.11 (-0.74, 0.95)	0.873
Smoking		
Smokers vs non-smokers	0.57 (-0.14, 1.28)	0.126
Alcohol consumption		
Alcohol consumption vs no alcohol	-0.57 (-1.37, 0.24)	0.152
General health		
Fair vs poor	0.63 (-0.88, 2.13)	0.553
Good vs poor	0.36 (-1.06, 1.78)	0.696
Psychological health		
Fair vs poor	-0.19 (-1.81, 1.43)	0.876
Good vs poor	-0.06 (-1.60, 1.49)	0.963
